# Supplementary material for: Neglected Spleen Transcriptional Profile Reveals Inflammatory Disorder Conferred by Rabbit Hemorrhagic Disease Virus 2 Infection
Source: Viruses. 2024 Mar 23;16(4):495. doi: 10.3390/v16040495 (PMC11054208; doi:10.3390/v16040495)
Supplement: Supplementary file 1 [file viruses-16-00495-s001.zip › Table S1.pdf]

Table S1. Sequences of the PCR primers used in this study.

Primers	Nucleotide sequence (5'–3')	PCR product length (bp)	Source
RHDV2-qF	TGGAACTTGGCTTGAGTGTTGA	127	This study
RHDV2-qR	ACAAGCGTGCTTGTGGACGG		
RHDV2-F	ACTACTAGCGTGGTCACCACC	481	Velarde et al., 2017
RHDV2-R	TTGTTATAAACGCTCAGGACCAAC		
IL1A-F	CTGCCATTGACCATCTTTC	253	This study
IL1A-R	TACTGCCACCACATTCTCC		
IL-6-F	GAGCATCCTGGAGACCATCAA	82	Delgado et al., 2019
IL-6-R	CCAGTGCCTCCTTTCTGTTCA		
IL-8-F	CCACACCTTTCCATCCCAAAT	122	Schnupf et al., 2012
IL-8-R	CTTCTGCACCCACTTTTCCTTG		
IL-22-F	GCAACAATCTCAGCCAATG	104	This study
IL-22-R	TCTCTCCACTCTCTCCAAGC		
CCL2-F	CTCCAGCATGAAGGTCTC	131	Li et al., 2014
CCL2-R	GCTCATTAGCCTCTTCACTG		
CXCL9-F	AATGAGGAATGGACGCTG	172	This study
CXCL9-R	TCACCTTGGTTGAATCCG		
NMUR1-F	ACAGGTGACCAAGATGCTG	167	This study
NMUR1-R	GAGCCGAGGTAGAAGAAGAC		
HSPB7-F	TCCACTCTGCCTCACTTAGG	186	This study
HSPB7-R	GAGACTCCACAGGTCAAAGG		
KCNIP2-F	TACCGAGGCTTCAAGAACG	130	This study
KCNIP2-R	GTTGGTGTCAAAGGCATTG		
GAPDH-F	TGACGACATCAAGAAGGTGGTG	120	Schnupf et al., 2012
GAPDH-R	GAAGGTGGAGGAGTGGGTGTC		
