# Supplementary figures and images for: Neglected Spleen Transcriptional Profile Reveals Inflammatory Disorder Conferred by Rabbit Hemorrhagic Disease Virus 2 Infection
Source: Viruses. 2024 Mar 23;16(4):495. doi: 10.3390/v16040495 (PMC11054208; doi:10.3390/v16040495)

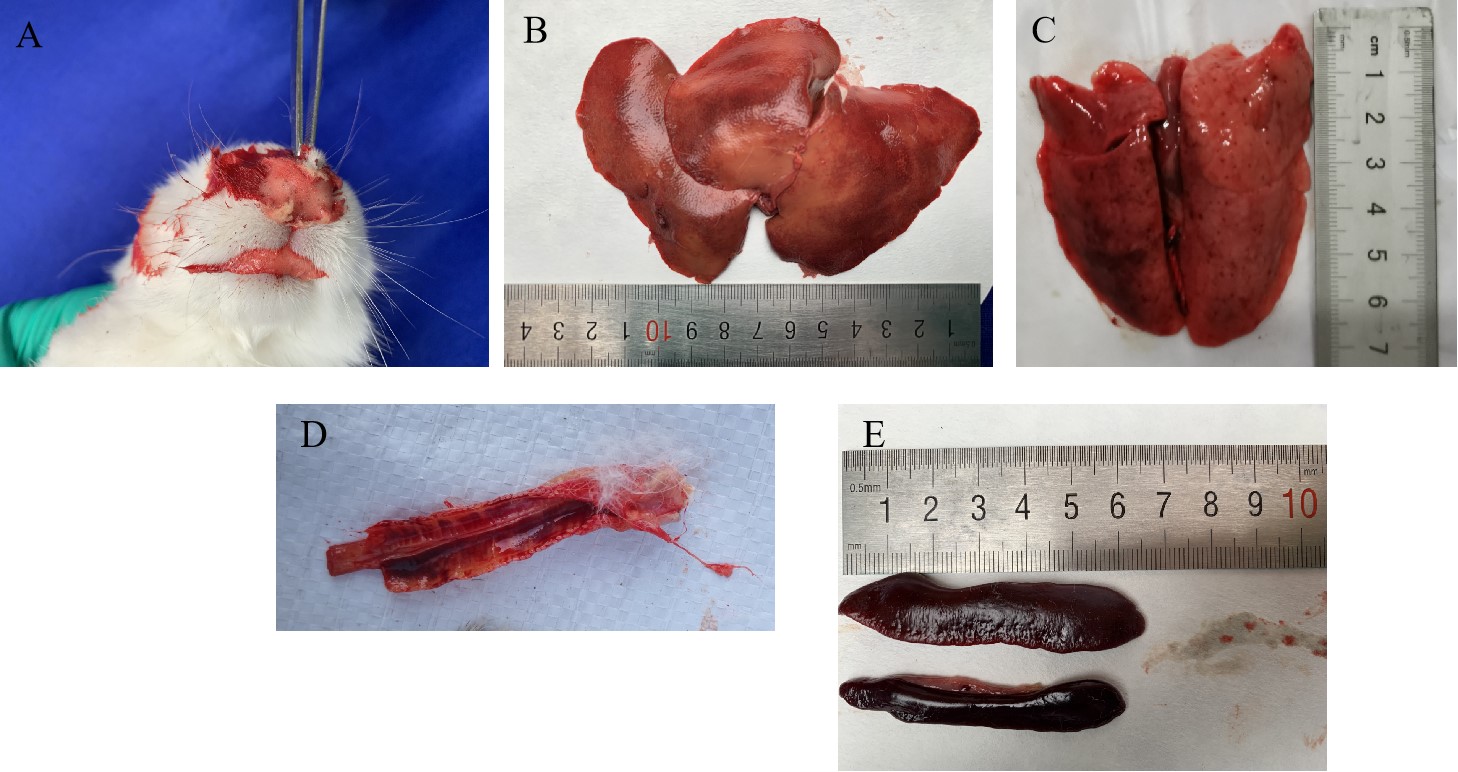

Supplement: Supplementary file 1 [file viruses-16-00495-s001.zip › Figure S1.jpg]

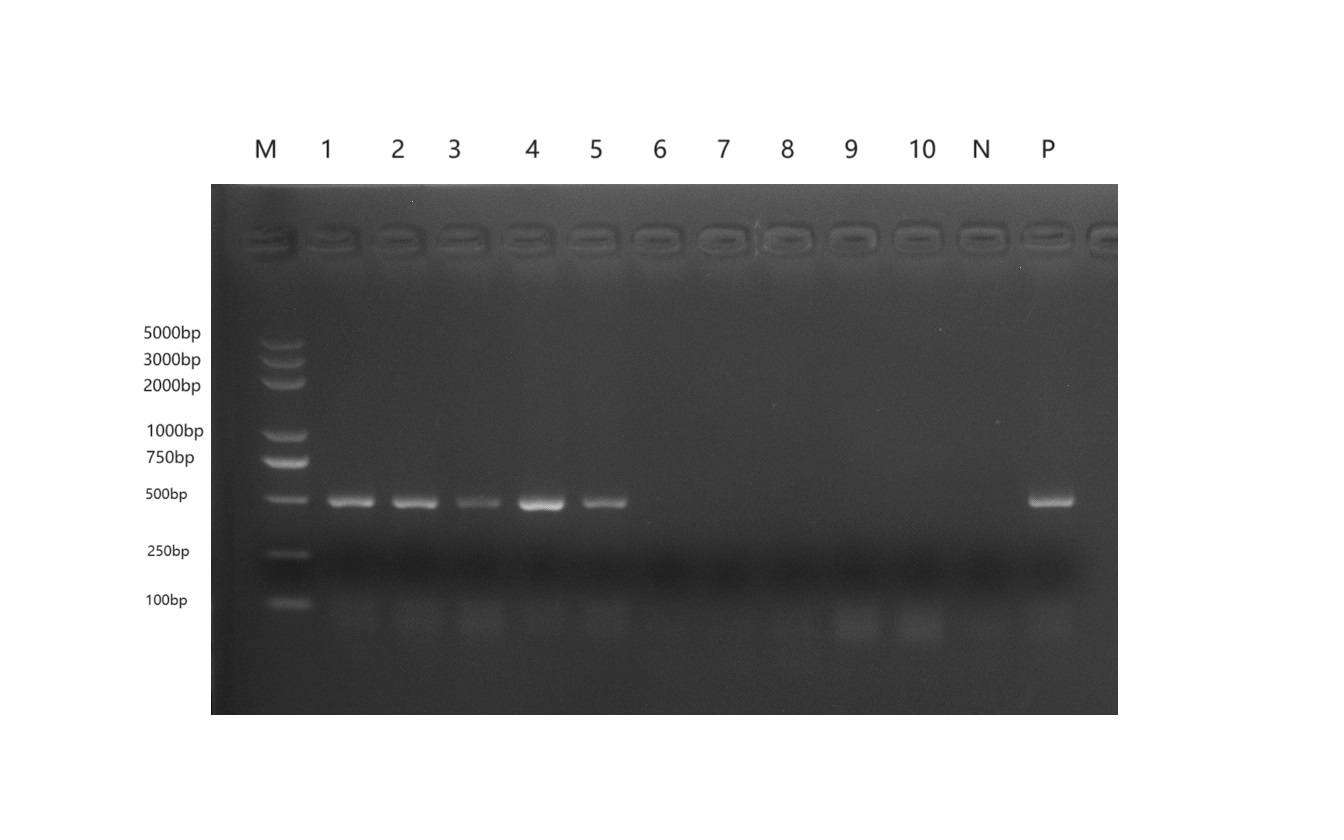

Supplement: Supplementary file 1 [file viruses-16-00495-s001.zip › Figure S2.jpg]

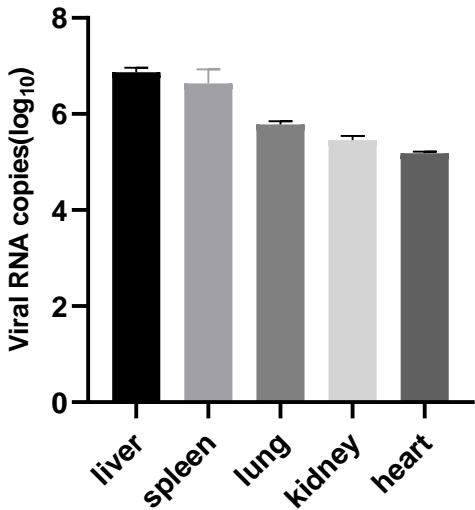

Supplement: Supplementary file 1 [file viruses-16-00495-s001.zip › Figure S3.pdf]

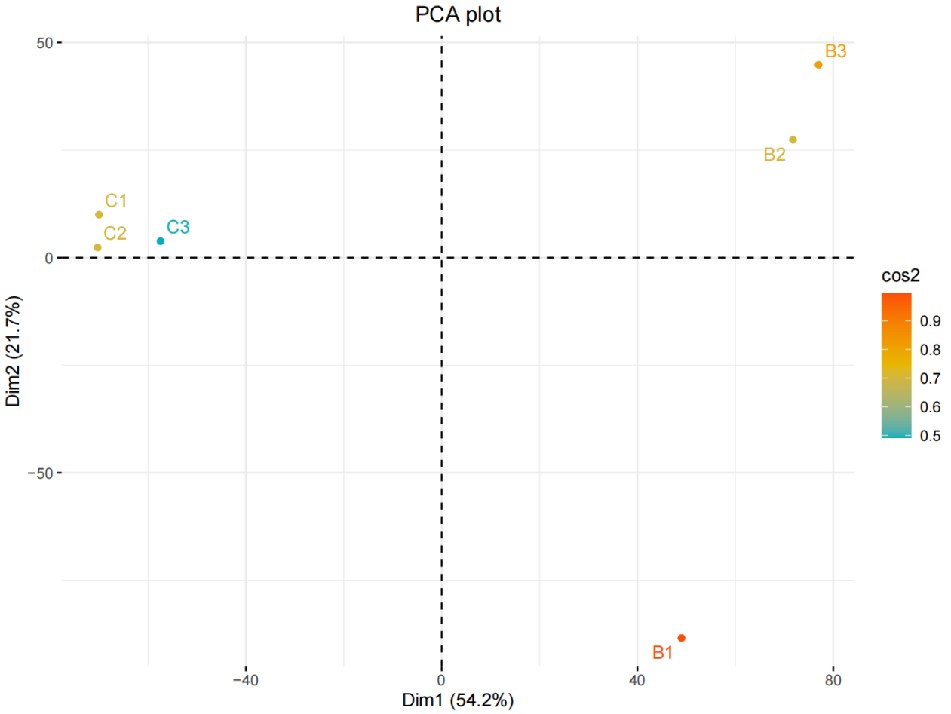

Supplement: Supplementary file 1 [file viruses-16-00495-s001.zip › Figure S4.jpg]
